# High activity of sequential low dose chemo-modulating Temozolomide in combination with Fotemustine in metastatic melanoma. A feasibility study

**DOI:** 10.1186/1479-5876-8-115

**Published:** 2010-11-10

**Authors:** Michele Guida, Antonio Cramarossa, Ettore Fistola, Mariangela Porcelli, Giuseppe Giudice, Katia Lubello, Giuseppe Colucci

**Affiliations:** 1 Department of Medical Oncology; National Institute of Cancer, Bari, Italy; 2 Department. of Radiology, National Institute of Cancer, Bari, Italy; 3 Department of Plastic and Reconstructive Surgery, University of Bari, Italy

## Background

Metastatic melanoma (MM) is an incurable chemoresistant cancer with poor prognosis. Until now, only few drugs have shown some activity. So this tumor represents an opportunity to verify new and more effective treatment strategies.

Presently, Dacarbazine (DTIC) remains the standard chemotherapy for MM with an overall response rate of approximately 10-15% with complete response in less than 5% of patients and a survival about 8-10 months [1,2]. No other agents have demonstrated better results than DTIC in phase III studies also when utilized as polichemotherapy or in association to immunotherapy [3-6].

Temozolomide (TMZ) has been recently utilized in MM. It is a novel oral alkylating agent having a high oral bioavailability and extensive tissue distribution, including penetration through the blood-brain barrier. Patients with MM achieved overall response rates of nearly 20% with single-agent TMZ as similar as DTIC [7-9]. Also nitrosureas are considered drugs of any activity in MM including patients with brain metastatic. Among nitrosurea analogs, fotemustine (FM) has been more extensively studied in MM, especially in Europe. It is a third generation chloroethylnitrosourea that has demonstrated significant antitumoral effects in MM with a response rate averaging 20%. However, its use is somewhat limited by its myelotoxic side effect, especially when old schedules are utilized [10-12].

The activity of alkylating agents depends on their capacity to form alkyl adducts with DNA, in some cases causing cross-linking of DNA strands. However, the antineoplastic activity of these agents is limited by cellular resistance principally induced by the DNA repair enzyme O(6)-methylguanine DNA-methyltransferase (MGMT), a DNA suicide enzyme which removes alkyl groups from alkylated DNA strands [13-16]. In tumor cell lines and xerografts an inverse correlation between the level of this protein and the sensibility to the cytotoxic effects of nitrosureas including FM has been demonstrated [17,18]. Moreover, studies evaluating the tumor MGMT levels in patients with brain tumors receiving nitrosureas reported a positive correlation between low level content of MGMT and a better survival [19,20].

Preclinical studies and recent clinical experiences also support the concept that continuous exposure to alkylating agent TMZ, streptozocin, procarbazine, and DTIC, can effectively deplete cells of MGMT, which is the primary mechanism of tumor resistance to nitrosureas, thus reversing the resistance to these chemotherapeutic agents [21-23]. In particular, sequential administration of TMZ and FM is able to induce depletion of MGMT both in blood lymphocytes and in tumoral tissue [24].

Recent clinical experiences have confirmed that continuous exposure to alkilating agent procarbazine in association with FM is an active treatment in patients with recurrent malignant gliomas [25]. At present, in spite of numerous experimental experience, very few data exist regarding the clinical use of TMZ as chemomodulating agent in MM patients. In particular, no established doses, timing and schedules are known.

Thus, we planned this study in MM patients to verify the hypothesis that depletion of MGMT induced by low dose TMZ could render melanoma cells more susceptible to FM. We used two different schedules of sequential combination of TMZ and FM in to assess their profile of toxicity and efficacy.

## Patients and methods

### Patients

Fourteen patients with histologically confirmed stage IV MM and chemotherapy-naïve were enrolled into two consecutive cohorts of 7 pts each, treated with two different schedules.

The patients were required to have measurable lesions (according to RECIST's criteria), adequate renal, hepatic and bone marrow functions, an adequate ECOG performance status (0-2) and life expectancy of at least 12 weeks. Adjuvant immunotherapy, and previous radiotherapy or locoregional treatments on non-target lesions were permitted. Patients with asymptomatic brain metastases were also enrolled if they had additional disease sites. Also patients with symptomatic brain metastases were admitted on condition that they had additional disease sites and brain disease stabilized by previous locoregional treatments. Patients who had received previous cytotoxic treatment for metastatic disease were not enrolled. The trial was approved by the local ethical committee and written informed consent was obtained from all patients before study entry.

The period of accrual was from April to December 2009. The main patient characteristic are listed in table 1. The median age was 64 years, range 38-76; ECOG PS 1, range 0-2. Disease sites included soft tissues/lymph nodes 13, lung 7, liver 3, bone 3, brain 1, spleen 1, adrenal gland 1, endopelvic mass 1. Basal LDH was evaluated in all patients (normal range 240-480 mg/dl). It resulted elevated in 1 patient (about double of the up limit of normal range) and near the upper normal limit in 3 patients. According to AJCC melanoma staging [2], 2 patients had M1a staging, 4 patients had M1b staging, and 8 patients had M1c staging. Two patients had only 1 metastatic site; 7 patients had 2 metastatic sites; 5 patients had 3 or more metastatic sites.

**Table 1:** Patient characteristics and clinical outcomes according to the two cohorts

N. Pts	Age (years)	Sex	ECOG PS	Primary site	DFI (months)	Basal LDH	N. cycle of chemotherapy	Disease sites	Response (duration)	Survival (months)
Cohort A										

1	69	F	1	Skin	4	360	7	Lung Soft tissue Lymph nodes	PR (9 months)	19+

2	76	M	1	Skin	2	238	6	Soft tissue	PR (7 months)	17+

3	49	M	1	Unknown	_	324	2	Lung Bowel	PR (6 months) CR (2 months)	14

4	73	M	2	Skin	96	379	1	Lung Brain	SD (4 months)	4

5	62	F	1	Anal mucosa	11	403	7	Endopelvic mass Lymph nodes	PD	9

6	71	M	1	Skin	36	291	6	Soft tissue Lymph nodes Bone	SD (5 months)	14

7	64	M	1	Scalp	7	251	2	Lung Bone	PD	13+

Cohort B										

1	64	M	1	Skin	10	474	3	Liver Lymph nodes Spleen Lung	PD	5

2	38	F	2	Skin	11	309	7	Soft tissue Adrenal gland Bone	PR (6 months)	10

3	76	F	1	Skin	25	289	7	Soft tissue Lymph nodes	SD (5 months)	12

4	48	F	1	Skin	24	394	6	Lung	SD (7 months)	14+

5	42	M	0	Skin	12	442	3	Liver Lung	PD	13+

6	75	F	1	Skin	12	839	8	Soft tissue Lymph nodes Liver	RP (11+ months)	13+

7	59	M	0	Skin	24	330	6	Lung Lymph nodes	SD (4 months)	13+

Abbreviations: LDH: lactate dehydrogenase; CR: complete response; PR: partial response; SD: stable disease; PD: progressive disease.

### Treatment

Two different treatment schedules were used for the two cohorts of patients. In the first cohort, TMZ was administered orally at a single dose of 100 mg/m^2 ^on days 1 and 2, 7 and 8; FM was given intravenously at a dose of 100 mg/m^2 ^on days 2 and 8, 4 h after TMZ. Treatment cycles were repeated every 4 weeks for 2 consecutive cycles and then every 3 weeks for further 6 cycles. In the second cohort of patients, chemotherapy was administered at the same dose but every 3 weeks for a total of 9 cycles.

Toxicity was evaluated according to the NCI-Common Toxicity Criteria grading system. Different grades of toxicity and eventual reduction of dose were evaluated before each cycle of therapy. Patients were assessable for toxicity if they had received at least one cycle of treatment. The FM dosage was reduced by nearly 25% of the starting dose when the severe (grade 3 or 4) hematologic toxicity occurred. A 50% dose reduction was required in case of severe (grade 3 or 4) new hematologic toxicity. Patients requiring more than two dose reductions and for whom dosing was delayed for up to 3 weeks were removed from the study. Drug administration was postponed by 1 week if there was no full hematologic recovery from the prior cycle of treatment. Granulocyte Colony Stimulating Factors (G-CSFs) were allowed after the patient experienced grade 3-4 neutropenia.

Patients with progressive disease (PD) at any time were withdrawn from the study. Patients with stable disease (SD) or with partial response (PR) or complete response (CR) continued the treatment according to the protocol.

### Evaluation

This study was designed to detect the toxicity and clinical response of two different schedules of sequential TMZ and FM association. The pre-study evaluation was completed within 2 weeks before receiving the study drugs. On entry, all patients had a complete medical history and physical examination. Complete blood cell count with differential and platelet count, serum lactate dehydrogenas and standard biochemical analysis were performed before every treatment cycle. A complete blood cell count was also performed every week to better studying the myelotoxicity of the treatment that is known being its principal dose-limiting toxicity. Before each cycle, common toxicity criteria, performance status and measurement of clinically assessable disease were documented. Patients were evaluated for response if they received one or more cycles of treatment. Tumor response was evaluated by physical examination, computed tomography scan, or other tests according to the basal evaluation performance or according to clinical requests.

Objective tumor response was evaluated according to Response Evaluation Criteria In Solid Tumors (RECIST) criteria. A complete response (CR) was defined as complete disappearance of all lesions. A partial response (PR) was defined as a ≥ 30% decrease in the sum of longest diameter of all measured lesions. Stable disease (SD) was defined as no significant change in measurable and nonmeasurable disease. Progressive disease (PD) was defined as a >20% increase in the product of the two longest perpendicular diameters of any measurable lesions or in the estimated size on nonmeasurable disease, the appearance of a new lesion, or the reappearance of old lesions.

In cohort A, patients performed the first re-evaluation after two cycles of therapy; then after every three cycles. In cohort B, patients were evaluated every three cycles of treatment.

## Results

### Safety and dose delivery

The toxicity profile was evaluated on 73 cycles of therapy delivered, 31 cycles for Cohort A (schedule 1-28) and 42 for Cohort B (schedule d1-21). The main side effects are reported in table 2. The schedule d 1,8-28 was characterized by a heavier hematological toxicity with respect to schedule 1-21, mainly in terms of thrombocytopenia G3-4 (3 of 7 patients *vs *1 of 7 patients). Nevertheless, platelet transfusions were not necessary and no clinically significant bleeding complications occurred. G3-4 neutropenia occurred in 1 patient in cohort A and in none in cohort B. G1-2 anemia frequently occurred in both cohorts of patients (in 4 and 5 patients respectively).

**Table 2:** Main side effects in the two cohorts of patients

**Cohort**	**Toxicity G3-G4**					**Toxicity G1-G2**			
		
	**Neutropenia**	**Thrombopenia**	**Anemia**	**Others**		**Neutropenia**	**Thrombopenia**	**Anemia**	**Others**
		
Cohort A Schedule 1,8,28	1/7 pts	3/7 pts	0/7 pts	0/7		4/7	4/7	4/7	4/7 (1 transaminase increasing; 3 nausea-vomiting)
		
Cohort B Schedule 1, 21	0/7 pts	1/7 pts	0/7 pts	0/7		6/7	5/7	5/7	5/7 (1 asthenia; 4 nausea-vomiting)

Other minor side effects included nausea-vomiting involving about 50% of patient in both cohorts, transaminase increase in 1 patient in cohort A, and asthenia in 1 patient in cohort B.

The median of delivered cycles was 5 (range 1-9). Dose reduction was necessary in 4 patients in cohort A and in 2 patients of cohort B due to severe thrombocytopenia. Chemotherapy was also delayed in 4 patients of cohort A and in 2 patients of cohort B because of failure of hematologic recovery prior next cycle of therapy.

### Response and survival

**Figure 1:**
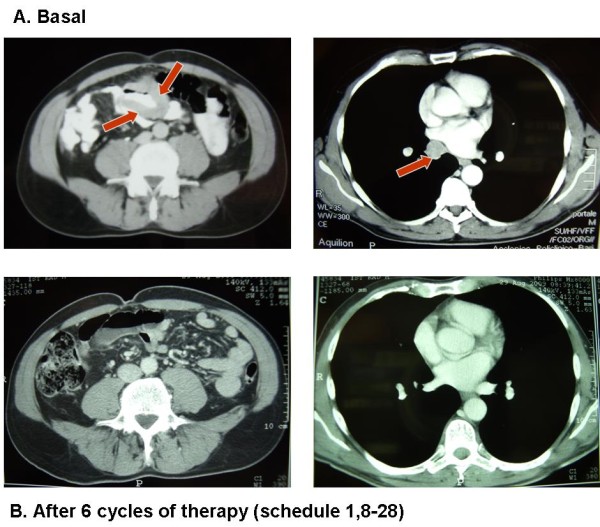
**Complete response in patient with mediastinal lymphopaty and bowel metastases treated in Cohort A (schedule d1,8-28)**.

Globally, we obtained 1 complete response (CR) and 4 partial response (PR) with a global response rate of 35.7%. The response duration ranged from 6 to 11+ months (median 8 months). We also obtained stable disease (SD) in 5 patients (35.7%), 2 in cohort A and 3 in cohort B. The unique CR lasting about 2 months occurred in a Cohort A patient who had mediastinal lymphopaty and bowel localizations (Figure 1). Than, after 8 months from starting therapy, patient presented an intestinal bleeding with a rapid anemization that required a surgical resection of part of the small intestine. The pathological analysis confirmed the diagnosis of metastases from melanoma. Patient died about 6 months later because of a rapid disseminated brain and meningeal spreading. The 2 PR occurring in Cohort A regarded one patient with multiple and diffuse cutaneous and subcutaneous lesions, and another patient with multiple disease sites including lung, lymph nodes and soft tissue. Both are alive after 19 months and 17 months, respectively. We also reported 2 SD in this group with a survival of 4 months in a patient with brain metastases who died for a cerebral hemorrhagic accident arising in the tumor metastasis. The other patient is died after 14 months.

**Figure 2:**
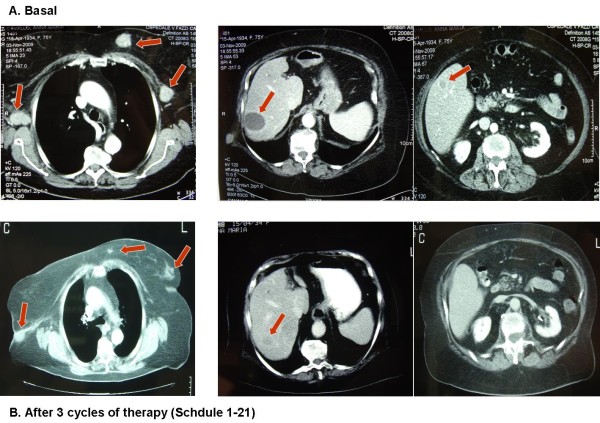
**Dramatic partial response in patient with liver, lymph nodes and subcutaneous metastases treated in Cohort B (schedule d1-21)**.

**Figure 3:**
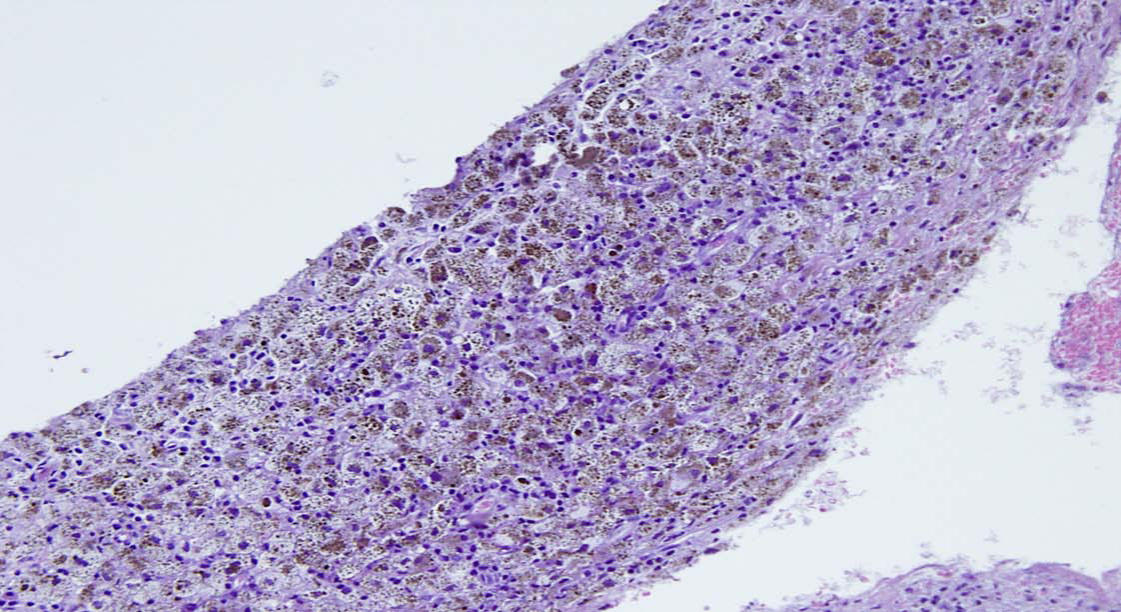
**Pathological features of a subcutaneous lesion biopsied after 3 cycles of therapy showing a diffuse regression of the neoplastic cells with abundant melanocitic pigment**.

**Figure 4:**
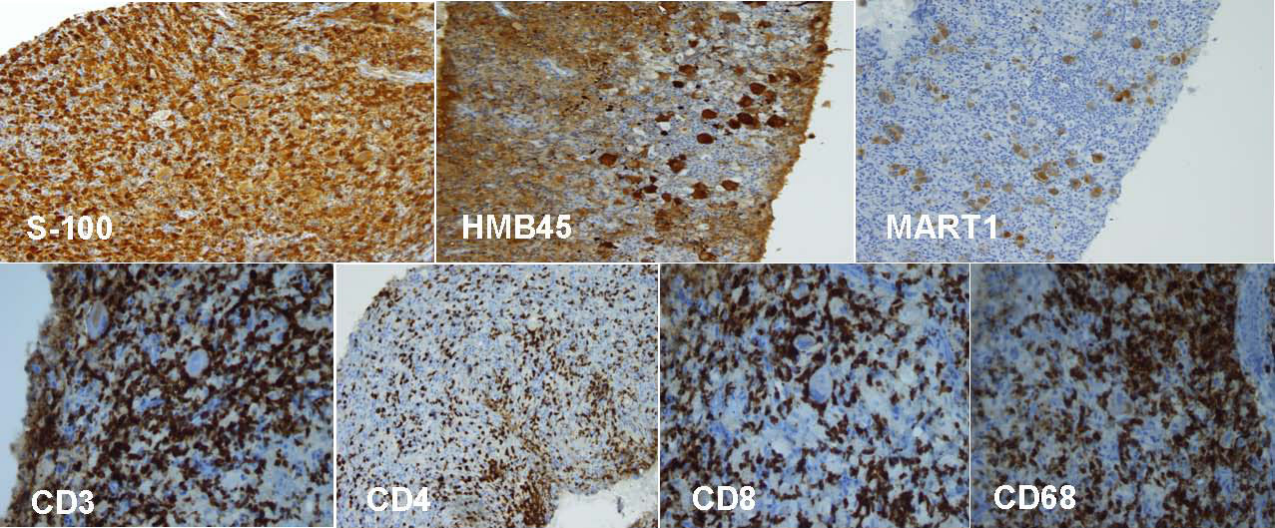
**Immunohistochemistry staining showed a strong positivity of the neoplastic component for S100 protein, HMB 45 and MART 1**. Moreover, an impressive lymphocytic (CD3+, CD4+, CD8+) and macrophage cells (CD68+) infiltration was present.

In the Cohort B we reported 2 PR and 3 SD. The PR regarded one patient with subcutaneous, adrenal gland and bone lesions. The duration of response was 6 months and the overall survival was 10 months. The other PR occurred in a female with a disseminated disease including axillaries lymph nodes involvement, diffuse subcutaneous localizations, multiple liver metastases, and elevated LDH levels. After 2 cycles of therapy patient showed a dramatic response in all metastatic sites (Figure 2) and a significant decrease of LDH. The biopsy of a subcutaneous lesion performed after the third cycle of therapy confirmed the diagnosis of metastatic melanoma and revealed a diffuse regression of the neoplastic cells with the presence of abundant melanocitic pigment. Immunohistochemistry revealed an intense staining of neoplastic component for S100 protein, HMB 45 and MART 1. Moreover, an impressive lymphocytic (CD3+, CD4+, CD8+) and macrophage cells (CD68+) infiltration was present (Figure 3, 4). At present, after 13 months from starting therapy, this patient is alive in PR. Regarding the 3 patients with SD, 1 died after 10 months and the others are alive after 13 months and 14 months. The median overall survival of the entire group is more than 13 months. At a median follow up of 13 months, 7 of 14 patients are alive.

## Discussion

This is the first clinical experience in MM using sequential non-therapeutic low dose TMZ previous full dose FM. We demonstrated that this is an active regimen in MM patients with an acceptable profile of toxicity. In fact, our preliminary data showed that as compared to TMZ or FM single agent, the sequential regimen of the two drugs together significantly enhances their antitumoral activity inducing high response rate and regression also in visceral sites as bowel and liver.

We used this sequential regimen to verify the hypothesis that continuous exposure to alkilating agent TMZ could effectively deplete tumoral cells of MGMT which is the primary mechanism of tumor resistance to nitrosureas. This hypothesis is supported by preclinical studies and clinical experiences [21-24]. Also recent experimental data in human melanoma cell lines have confirmed the presence of a close correlation between MGMT activity and the level of resistance to TMZ and FM, although a wide variability in MGMT activity among different cell lines was noted [26]. The Authors also reported that the MGMT inactivation by O(6)-benzylguanine sensitized all melanoma cell lines expressing MGMT to TMZ and FM-induced apoptosis. Moreover, the MGMT transfection attenuated the apoptotic response, supporting the hypothesis that O(6)-alkylguanines are critical lesions involved in the initiation of programmed melanoma cell death [26].

Further clinical experiences carried out in patients with recurrent cerebral tumons confirmed that continuous exposure to alkilating agent procarbazine in association to FM is an active therapeutic option for patients with glioblastoma heavily pretreated [25].

Regarding the association of TMZ and nitrosureas in MM patients, only two studies have been published in which TMZ was used in association to lomustine [27] and FM [28], respectively. In both studies full doses of both drugs were utilized with an therapeutic additive/synergistic intent. Nevertheless, despite of a high response rate, an unacceptable toxicity was reported with myelotoxicity being the principal dose-limiting toxicity. In particular, the study of Tas et al [28] reported a response rate of 35%, but the median survival was only 6.7 months with a dose reduction in the 45% of patients, a dose delay in 32,5%, and an early treatment discontinuing in 27,5%. Notably, in our study we report a response rate of 35.7% and a stable disease in 35.7% of patients with a survival over 13 months.

At present, very few data regarding the use of low dose TMZ as chemomodulating agent are available and no established doses and schedules exist [22,24]. Also the interval between the two drugs administration is not clear. Some Authors have reported that the administration of TMZ divided over two consecutive day at the dose of 100-200 mg/m^2 ^per day, seems to induce a substantial MGMT depletion at the time of FM administration given in the second day about 4 hours after TMZ [24,25]. Nevertheless, a wide inter-individual variation and no definitive data are available.

So, we used two schedules of TMZ and FM (day 1,8-21 *vs *day 1-21) in two well balanced cohorts of 7 patients each, to identify the regimen that better conciliates antitumor activity with an acceptable toxicity. In according to the data previously reported [24,25], we administered TMZ at 100 mg/m^2 ^per two days and FM at 100 mg/m^2 ^in the second day 4 hours after TMZ. We reported high response rate with this regimen in both cohorts of patients and a disease regression also in visceral sites and in patients with multiple metastatic localizations. Globally, we obtained an overall response of 35,7% with 1 CR and 2 PR in cohort A (regimen d1,8-28) and 2 PR in cohort B (regimen d 1-21). Five SD were also reported (35.7%), 2 in cohort A and 3 in cohort B. The median overall survival of the entire group was over 13 months. At this time, 7 of 14 patients are alive yet.

The unique CR occurred in a Cohort A patient with mediastinal lymphopaty and bowel localizations lasting about 8 months (Figure 1). The 4 PR occurred in patients with multiple and diffuse disease including lung, liver, bone, adrenal gland, lymph nodes and soft tissue. The unique patient with brain metastases died after 4 months of SD because of a cerebral hemorrhagic accident arising in the metastatic lesion without evidence of disease progression.

Interestingly, one PR in cohort B, occurred in a female with a disseminated disease involving axillaries lymph nodes, diffuse subcutaneous lesions, and multiple liver metastases. After 2 cycles of therapy the patient showed a dramatic response in all metastatic sites (Figure 2). At present, after 13 months from starting therapy, this patient is alive in PR. The biopsy of a subcutaneous lesion performed after the third cycle of therapy confirmed the diagnosis of metastatic melanoma and evidenced a diffuse regression of the neoplastic cells with abundant melanocitic pigment. Immunohistochemistry staining showed a strong positivity for melanoma associated antigen S100 protein, HMB 45 and MART 1. Surprisingly, an impressive lymphocytic (CD3+, CD4+, CD8+) and macrophage cells (CD68+) infiltration was also present (Figure 3, 4) meaning that immunomediated mechanisms have been also burst after TMZ-FM treatment, probably due to the massive disruption of neoplastic cell and consequent deliverance of tumoral associated antigens.

Regarding the toxic profile, there was a significant difference between the two cohorts of patients, principally in terms of myelotoxicity. In particular, the d1,8-28 schedule was characterized by a heavier G3-4 thrombocytopenia (3 of 7 patients) with respect to the d1-21 regimen (1 case of G3 thrombocytopenia) with a dose reduction in 4 patients in cohort A and in only 2 patients in cohort B, and chemotherapy delayed in 4 patients in cohort A and in 2 patients in cohort B. In summary, the d1-21 schedule resulted similar to the 1,8-28d schedule in term of activity, but it was superior in terms of tolerability and manageability guarantying the dose and timing planned.

Of course, an attempt to correlate the basal level of MGMT as well as the measurement of its depletion during therapy could permit to distinguish responder from non-responder patients. Nevertheless, this was not an objective of present study. In fact, our purpose was to evaluate the feasibility, tolerability and the activity of this new treatment. The study of the correlation between MGMT level and clinical outcomes has been planned in our ongoing phase II study.

## Conclusions

In the current study we demonstrated that the sequential combination of low dose TMZ and FM has a high activity in MM patients with an acceptable toxicity. The 1-21d schedule showed similar activity and a better toxic profile with respect to the 1,8,28d schedule; thus, we are using the 1,21d schedule in our phase II ongoing study aiming to confirm the high activity of this association in MM patients.

## Competing interests

The authors declare that they have no competing interests.

## Authors' contributions

MG carried out the study design and drafted the manuscript. AC cured the radiologic valuations. EF participated in the design of the study and performed the statistical analysis. MP participated in the study design and helped to draft the manuscript. GG participated in the patient accrual. KL participated in the preparation of the manuscript. GC carried out the coordination of the study and drafted the manuscript.

All authors read and approved the final manuscript.
